# Dye Tracking Following Posterior Semicircular Canal or Round Window Membrane Injections Suggests a Role for the Cochlea Aqueduct in Modulating Distribution

**DOI:** 10.3389/fncel.2019.00471

**Published:** 2019-10-30

**Authors:** Sara Talaei, Michael E. Schnee, Ksenia A. Aaron, Anthony J. Ricci

**Affiliations:** ^1^Department of Otolaryngology-Head and Neck Surgery, Stanford University School of Medicine, Stanford, CA, United States; ^2^Department of Molecular and Cellular Physiology, Stanford University School of Medicine, Stanford, CA, United States

**Keywords:** drug delivery, cochlea aqueduct, inner ear, perilymph, endolymph, round window, posterior semicircular canal

## Abstract

The inner ear houses the sensory epithelium responsible for vestibular and auditory function. The sensory epithelia are driven by pressure and vibration of the fluid filled structures in which they are embedded so that understanding the homeostatic mechanisms regulating fluid dynamics within these structures is critical to understanding function at the systems level. Additionally, there is a growing need for drug delivery to the inner ear for preventive and restorative treatments to the pathologies associated with hearing and balance dysfunction. We compare drug delivery to neonatal and adult inner ear by injection into the posterior semicircular canal (PSCC) or through the round window membrane (RWM). PSCC injections produced higher levels of dye delivery within the cochlea than did RWM injections. Neonatal PSCC injections produced a gradient in dye distribution; however, adult distributions were relatively uniform. RWM injections resulted in an early base to apex gradient that became more uniform over time, post injection. RWM injections lead to higher levels of dye distributions in the brain, likely demonstrating that injections can traverse the cochlea aqueduct. We hypothesize the relative position of the cochlear aqueduct between injection site and cochlea is instrumental in dictating dye distribution within the cochlea. Dye distribution is further compounded by the ability of some chemicals to cross inner ear membranes accessing the blood supply as demonstrated by the rapid distribution of gentamicin-conjugated Texas red (GTTR) throughout the body. These data allow for a direct evaluation of injection mode and age to compare strengths and weaknesses of the two approaches.

## Introduction

Inner ear end organs contain the sensory epithelium for balance and hearing. These fluid filled compartments are exquisitely sensitive to motion, particularly the fluid motion within these end organs (Hudspeth, 1989). Understanding fluid flow and compartmentalization within these systems is important at multiple levels. The ionic environment surrounding the sensory hair cells is critical to function (Wangemann and Schacht, 1996). Many pathologies associated with hearing loss and balance disorders stem from disruption of these fluid compartments or the pressure associated with these compartments [e.g., Meniere’s disease (Hornibrook and Bird, 2016), Superior Semicircular Canal (SCC) Dehiscence (Minor et al., 1998), and Benign Paroxysmal Positional Vertigo (BPPV) (Vazquez-Benitez et al., 2016)]. Finally, therapies to prevent damage and to repair or replace damaged tissue will rely on our ability to deliver compounds uniformly and selectively to the inner ear compartments and end organs that require treatment. We cannot adequately design and assess therapeutics if we do not understand how the delivery system impacts drug distribution both within the ear but also to the brain and beyond.

RWM injection is a common mode of compound delivery that provides direct delivery into the perilymphatic space (Liu et al., 2005, 2007; Iizuka et al., 2008; Akil et al., 2012; Askew et al., 2015; Plontke et al., 2016; Dai et al., 2017; Landegger et al., 2017; Pan et al., 2017; Yoshimura et al., 2018). Plontke et al. (2016) showed direct intracochlear injection into the RWM of guinea pigs resulted in a basal–apical gradient in distribution of the injected markers (i.e., fluorescein or fluorescein isothiocyanate-labeled dextran). RWM injections in guinea pigs lead to compound detection in subarachnoid space, presumably traveling through the nearby cochlea aqueduct (Kaupp and Giebel, 1980). Recently, rapid detection of a biological dye in spinal cord and brain of mice after RWM injection was reported (Akil et al., 2019).

PSCC is another site for inner ear injections that offers the advantage of easy access with limited middle ear damage (Kawamoto et al., 2001; Suzuki et al., 2017; Isgrig and Chien, 2018). Data from lateral SCC (LSCC) injection of markers and dexamethasone suggest a more uniform distribution within the cochlea of guinea pigs (Salt et al., 2012a).

Here we directly compare RWM and PSCC injections at two ages in mice using similar volumes and flow rates. These experiments normalize for experimental variability as well as species and allow for direct assessment of route of entry and age. We demonstrate more uniform dye delivery using PSCC injections with less dye appearing in brain than with RWM injections. RWM injections result in more dye in the brain, implicating the cochlear aqueduct as a potential shunt to cochlear drug flow. Finally, GTTR injected in the PSCC resulted in dye appearing throughout the body, suggesting it can rapidly enter the blood supply, likely by crossing the membranous labyrinth membranes. Together these data begin to identify key parameters regulating distribution of compounds within inner ear compartments.

## Materials and Methods

Injections of trypan blue (4.6 mM, Life Technologies Corporation, United States), Methylene Blue (100 mM, ACROS Organics, United States), and GTTR [270 μM (Myrdal et al., 2005)] were made directly into the inner ear of mice. For *ex vivo* imaging, the dissected tissue was kept in Hanks’ balanced salt solution (HBSS, Life Technologies Corporation, United States) buffer. C57/BL6 mice of both sexes at postnatal days of P1–P3 and P21–P23 were used for these studies. All animal procedures were approved by the Animal Care and Use Committee at Stanford University.

### Injection Protocol

One microliter of compound was injected at a flow rate of 300 nl/min to five to six animals per group and the dye presence at the cochlea base, middle, and apex was monitored at different time points. Cochlear perilymphatic space for an adult mouse was previously estimated as 0.62 μl (Thorne et al., 1999). In this study, we chose a 1 μl injection volume for all experiments to provide enough dye for a complete replacement of the cochlear perilymph. No change in injection volume was used to compensate for any changes in inner ear volume with age. We tested several dyes including methylene blue, AM1-43 (100 μM, Biotium, United States), trypan blue, and GTTR. We chose to use trypan blue as the main dye for these studies because it interacted least with the tissue, it is not taken up by the cells (like AM1-43, methylene blue, or GTTR), nor does it appear to stick to the tissue (like methylene blue). 300 nl/min was selected as the fastest time that did not disrupt the tissue, we were finding a balance between going quickly to limit movement issues with the pipette placement and limiting any damage that might be caused by the fluid pressure inside the cochlea during injection. Flow rate was maintained regardless of age and injection site.

In all experiments, a 10 μl gas-tight syringe (Hamilton, United States) was mounted onto a microinjection pump [UMP3 UltraMicroPump, WPI, United States (Salt et al., 2012a)] and coupled to a glass micropipette tip. The rate and duration of injection were controlled by a microprocessor-based controller (Micro 4, WPI, United States) affixed to the microinjection pump to ensure the appropriate injection volume. The syringe and the micropipette were first filled manually with phosphate buffer saline (PBS) up to 4 μl of the syringe volume. After mounting the syringe onto the pump, using the Micro 4 controller, the pump was programmed to load a 1 μl air gap at the micropipette tip via suction, before loading the dye. Four microliters of the dye was then suctioned into the micropipette, leaving an air gap between PBS and the dye. This approach limited compression, allowing for a highly reproducible volume injection. The glass micropipettes were generated with a micropipette puller (Sutter Instrument Co., Model P-97, United States) and then scored and broken to ∼25 μm inner diameter and ∼45 μm outer diameter tips. Similar pipettes were used for both neonatal and adult injections. For precise localization of the micropipette tip during injections, the pump was mounted on a motorized manipulator (Exfo Burleigh, PCS-6000, United States).

### Data Collection

M320 F12 ENT Microscope with a fully integrated camera (Leica, movie resolution: 720 × 480 pixels, image resolution: 2048 × 1536 pixels, Germany) was used for the surgical procedures and live imaging of the dye distribution within the cochlea. The integrated camera stored images and videos on a secure digital (SD) memory card. Example videos are presented in the Supplementary Material S1–S4. The injection process, starting at time 0, was video recorded in an MP4 format and converted to 8-bit JPEGs at 30 s intervals. Free Video to JPG Converter (V.5.0.101^1^) was used for converting the MP4 files. Following completion of the infusion, JPEG images (8-bit) were captured at 3-min intervals (from 4 to 34 min). SZX10 microscope (Olympus, Japan) equipped with CCD color and monochrome cameras (QIclick-F-CLR-12 and QIclick-F-M-12, 1392 × 1040 pixels, 6.45 μm^2^ pixel size, Teledyne QImaging, Canada) was used for dissections and imaging of the dissected cochleae and brains at the ∼5 min and 1-h time points as well as for the fluorescent images using GTTR. Images with TIFF (12-bit) and JPEG (8-bit) formats were captured.

### Data Analysis

All images were analyzed similarly. Intensity was measured in 50 (*in vivo* images) and 100 μm (*ex vivo* images) diameter regions in apex, middle, and base. For all intensity measurements, Fiji software (open source image processing package^2^) was used. All statistics were performed with Origin Software using two-sample or pair-sample *t*-tests. For *in vivo* images, data from apex, middle, and base from each cochlea were pooled for group comparisons if no regional differences were observed.

### Computational Modeling

Finite element method (FEM) simulation software (COMSOL Multiphysics 5.3a) was used for computing the velocity magnitude along the perilymphatic compartment, during injections via PSCC and RWM. The 2D Laminar Flow Module for incompressible flows was applied to solve Navier–Stokes equations coupled with the continuity equation for conservation of mass in the periymphatic compartment. The perilymphatic compartment was modeled as a 2D pipe with three connected sections representing scala tympani (ST), scala vestibuli (SV), and SSCs. As shown in Figure 9C, based on the measurements reported previously for an adult mouse (Thorne et al., 1999), 4.55 and 3.98 mm were chosen for the length of ST and SV. For simplicity, the width of each section was kept constant along the length (i.e., SV: 200 μm, ST: 140 μm). A 4 × 0.09 mm^2^ section was added to represent SCCs. The cochlea aqueduct was assumed to have a 100 μm width opening in ST, at 650 μm distance from the RW. It was assumed that the injection was directly into the perilymphatic space, and no back flow was considered at the injection sites. The injection sites at PSCC and RWM were both 25 μm located in the middle length of SCC and middle width of ST, respectively. During the injection, the only inlet for the system was the injection site. In our experiments that we injected the dye at 300 nl/min through a micropipette with 25 μm diameter, the flow velocity at the micropipette tip was calculated 0.01 m/s which was chosen as the inlet velocity in our model. We first assumed that the perilymphatic space inside the inner ear is an isolated compartment with only one outlet (i.e., cochlea aqueduct) and one inlet (i.e., injection site) (Figure 9D). No material exchange through the walls was possible with this assumption. In our second model (Figure 9E), we defined a distributed leaky wall at the top of all compartments at which the fluid velocity during the injection was larger than zero (e.g., 10^–5^ m/s). This leak is meant to represent both biological and experimental leak associated with the procedure.

### Neonatal Surgeries

Neonatal mice were anesthetized with hypothermia induced by placing the pups on a nitrile glove sitting on crushed ice for 3–5 min. Hypothermia was approved by Administrative Panel on Laboratory Animal Care (APLAC) and Stanford veterinary staff for use in neonatal animals. The animals were transferred to an ice water cooled aluminum block to perform the surgery. A 3–5 mm incision was made behind the ear, in the postauricular region with microscissors; muscle tissue was removed with forceps to expose the PSCC and bulla. Kimwipes (Kimtech, United States) were used for absorbing blood at the incision site. For the experiments where cochlea dye distribution was continuously recorded during and after the injection, a larger incision was made in the postauricular region (8–10 mm). The auricle, tympanic membrane, and bulla were removed while the stapes bone was left intact. This approach allowed for better viewing of the cochlea (shown in Figure 2). For PSCC injections, the micropipette was inserted into the PSCC using a micromanipulator. The bony labyrinth in neonatal mice was soft enough to allow micropipette tip penetration. The micropipette tip was visually assessed before and after each injection, and broken tips were not used for data collection. From this experiment it was not evident if the tip ruptured the membranous labyrinth, so it is possible that both endolymph and perilymph compartments were injected. As discussed by other groups, it is possible that the membranous labyrinth was ruptured during PSCC injections potentially causing direct access to the endolymphatic space (Kawamoto et al., 2001; Isgrig and Chien, 2018; Yoshimura et al., 2018). At the end of infusion and immediately after pulling out the injection pipette, a small droplet of 101 cyanoacrylate adhesive (Permabond, United States) was applied to the injection site, which was then covered with a muscle patch. For the RWM injections, the initial incision was made using microscissors at a position about 2 mm lower than the one in PSCC injections in order to expose the bulla. A small opening (1–2 mm) was made in the bulla to expose the RW, and the glass micropipette was inserted into the RWM. At the end of infusion, the injection pipette was removed and the RW was covered with a plug of muscle, and a droplet of cyanoacrylate was applied on the top to attach the muscle plug to the RW niche. For all neonatal surgeries, after sealing the injection site, the surgical incision was closed, and sealed with surgical glue (Suturevet Vetclose, Henry Schein Animal Health, United States). The entire procedure was accomplished within 15–20 min. For the experiments in which the cochleae were examined at 1-h post injection, pups were kept under hypothermia for the duration of the surgery and then transferred to a heating pad (37°C). Pups revived within 3–5 min. Animals were euthanized at 1-h time point for further evaluation.

### Adult Surgeries

Adult mice weighing between 8.5 and 10.5 g were anesthetized with an intraperitoneal injection of a mixture of ketamine (100 mg/kg) and xylazine (10 mg/kg). While animals were anesthetized, the surgery was performed on a heating pad at 37°C. The fur behind the left ear was removed using hair removal cream and sterilized with 10% povidone iodine followed by 70% ethanol. A postauricular skin incision of ∼10 mm was made. To expose the cochlea for live imaging of the dye distribution during injection, a posterior transcanal incision was made with microscissors. Twelve and six o’clock medial cuts were then made in order to transect the remainder of the ear canal and to excise the bulla. The canal cartilage, dorsolateral surface of the auditory bulla, tympanic membrane, and ossicular chain were then removed except for the stapes bone allowing visualization of all cochlea turns (from the top view as shown in Figure 2), the oval window (OW), and round window (RW). All hemostasis was achieved with electrocautery (Medline, United States). After this exposure, for the PSCC injection, the sternocleidomastoid muscle was cut proximally, while the muscles covering the temporal bone were separated and retracted dorsally to expose the bony wall of the PSCC. A chemical canalostomy was achieved by applying 36.2% phosphoric acid etching gel (Young, United States) with the same caliber tip of the glass micropipette as used for injection and 20 s were allowed to take effect for bone resorption, and enough time to leave endosteum covered by a thin layer of bone (Alyono et al., 2015). A moist roll of cotton was used to remove the remaining gel. The etching treatment softened the bone enough to insert the perfusion micropipette. Using the micromanipulator, the tip of the glass micropipette was advanced (∼2–3 mm) into the canal angled toward the ampulla. This assured no backflow of the injected material and a sturdy tip insertion. The injection of trypan blue was performed as above. After removing the micropipette, the hole was plugged with small pieces of muscle, and covered with 101 cyanoacrylate. For the RWM injection, after the cochlea exposure was achieved, injection was performed with a glass micropipette into the RW niche passing through the RWM. All injections were performed and recorded under the surgical microscope described earlier and the delivery site was closely monitored for leakage during all procedures. No dye leakage was observed in the neonatal and adult mice during PSCC injection. In some RWM injections (∼10% of neonates and ∼50% of adults) a leakage was observed from the injection site. The higher leakage rate in adult may be due to the increased intracochlear pressure compared to neonatal animals which resulted in more backflow. Those animals were not included in the study. After removing the micropipette, the RW was sealed with a plug of muscle. For the experiments where the bulla was removed and the cochlea was monitored during the injection for up to 30 min post injection, the imaging region was occasionally covered by blood or other fluids during the imaging. When removing of these fluids could not be done properly, the animal cases were removed from the data analysis. At the end of each experiment, animals were euthanized under anesthesia. Mouse temporal bones and each side of the brain were harvested and placed in HBSS after which the tissue was imaged.

## Results

**FIGURE 1:**
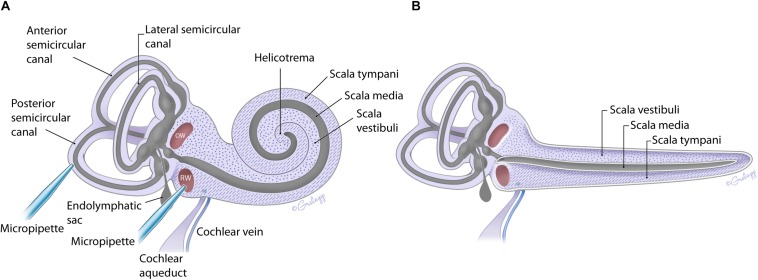
Schematic of the mouse inner ear with coiled **(A)** and uncoiled **(B)** configurations of the cochlea. The fluidic compartments for endolymph and perilymph are depicted in gray and light purple, respectively. The cochlea compartment of scala tympani is indicated with the hatch markings and scala vestibuli with the dots. All other compartments are as labeled. The approximate location of the injection pipette is illustrated for PSCC and RWM.

The fluid filled inner ear consists of discreetly positioned sensory epithelium of the vestibular and auditory systems housed in a contiguous membranous compartment (Sterkers et al., 1982; Hudspeth, 1989). These end organs are located within the temporal bone and are protected by a bony capsule (Felten et al., 2016). The bony capsule is filled with perilymph, a solution very similar to cerebral spinal fluid, and surrounds a membranous labyrinth containing endolymph, a high potassium, and low calcium solution created by supporting cells within each end organ (Sterkers et al., 1988; Wangemann and Schacht, 1996). The endolymphatic compartment is located within the perilymphatic space as shown in Figure 1 (Sterkers et al., 1988; Forge and Wright, 2002; Salt et al., 2012a; Felten et al., 2016). The perilymph and endolymph solutions are shared by the vestibular and cochlear end organs, yet the fluid composition is often different between end organs (Sterkers et al., 1988; Wangemann and Schacht, 1996). The mammalian cochlea has three chambers, ST and SV contain perilymph, and scala media (SM) contains endolymph (Figure 1). The SV contacts the OW membrane (OWM) and the ST contacts the RWM (Fernández, 1952; Salt et al., 2012a, b; Felten et al., 2016). SV is contiguous with ST, converging at the apex, at the helicotrema (Fernández, 1952; Salt et al., 1991; Felten et al., 2016; Wright and Roland, 2018). At the basal end the SV is contiguous with the vestibule while the ST ends at the RWM (Figure 1). The cochlear aqueduct is a small channel extending from the ST at the cochlea basal turn, adjacent to the RWM that projects into the cranial cavity (Schuknecht and Seifi, 1963; Gopen et al., 1997). The function and patency of the cochlear aqueduct remains unclear but is suggested to regulate pressure (Carlborg, 1981; Carlborg et al., 1982). The endolymphatic sac extends from the vestibule between the cochlea base and the vestibular end organs and similarly may be a reservoir for excess endolymph production (Kimura and Schuknecht, 1965; Couloigner et al., 1999). Although the inner ear contains two independent fluid compartments, flow through these compartments is complex due to the multiple routes and different resistant properties offered by the shape of the various end organ components. The potential for ion transport between compartments further complicates our understanding of compound delivery (Salt et al., 1991; Salt et al., 2012b).

In the present work, flow within the inner ear was visualized by injecting trypan blue at two independent sites indicated in Figure 1A. The impact of age on the dye distribution within the cochlea after a PSCC or RWM injection was studied by applying similar injection protocols to neonatal and adult mice and comparing the results.

**FIGURE 2:**
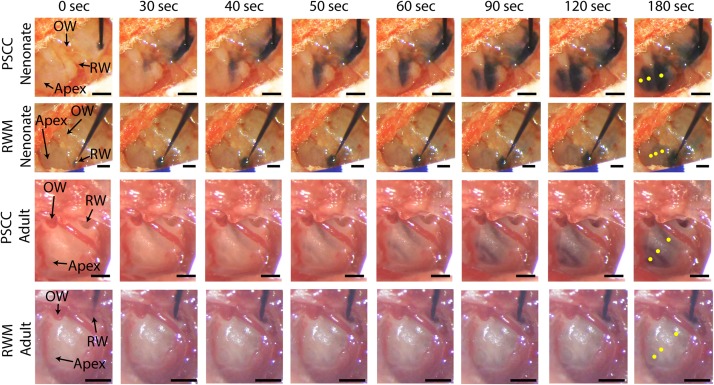
*In vivo* monitoring of trypan blue dye distribution along the cochlea during PSCC and RWM injection in neonatal and adult mice. In all experiments, the microinjection pump was turned on at 0 s injecting 1 μl of the dye at 300 nl/min. The regions of interest where the intensity was measured at **apex**, **middle**, and **base** for each experiment are shown in yellow in the last picture of each row (180 s). Scale bar is 500 μm. The *X*-axis provides time snapshots and the *Y*-axis the specific group being injected.

Figure 2 presents *in vivo* images used for analysis of the dye distribution within cochlea for each experimental group. The images show dye progression within the cochlea at different time points during the injection. From the imaging angle shown in these pictures, the distribution of the dye upon PSCC injection was visible in the cochleae of both neonatal and adult mice. The dye first appeared at the OW region (see Figure 2 PSCC neonate time 30 s as example), continued to the cochlea base, and then migrated toward the apex. Although we did not have the resolution to differentiate migration in ST and SV, the later appearance of the dye at RW region (after reaching the apex) suggests that the dye had first entered the SV and then continued into the ST compartment as was reported in the literature (Salt et al., 2012a). The dye distribution pathway in the cochlea upon RWM injection was not as clearly visible at this imaging angle (see alternate *ex vivo* images in Figure 5). Despite this difficulty we could track dye in each configuration but cannot compare absolute intensity values between ages for this experiment. In contrast to PSCC injections, visual inspection of RWM injections, *ex vivo*, show dye accumulation at the cochlea base with less dye found in middle turn and virtually no dye seen at the apex.

**FIGURE 3:**
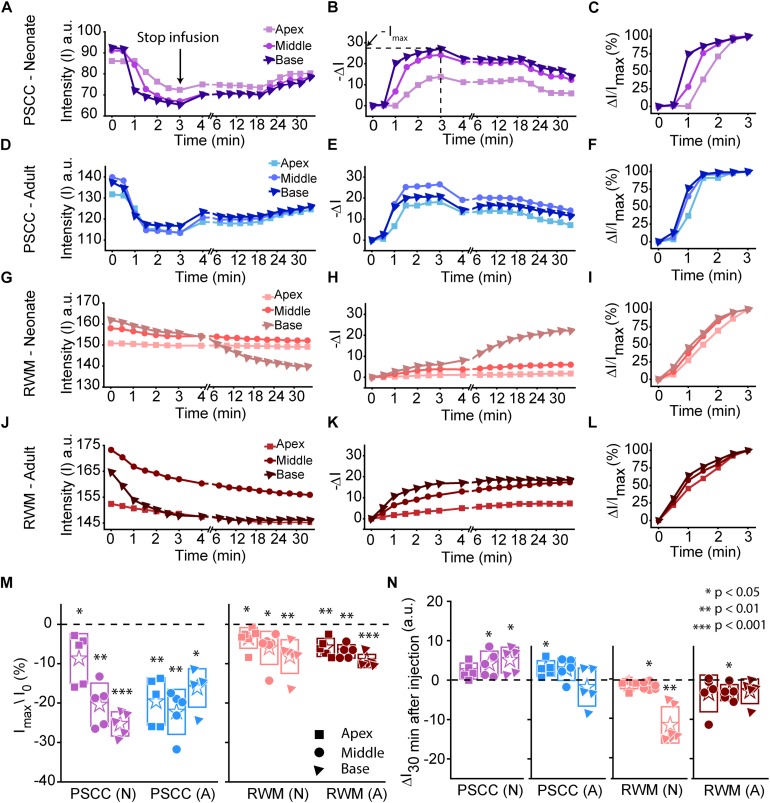
Representative plots obtained from one animal in each experimental group (*n* = 5, 6 in each group) showing dye distribution along the cochlea during PSCC and RWM injection in neonatal and adult mice from starting the pump up to 34 min. Injection time was from 0 to 3 min. Graphs **(A,D,G,J)** show the raw intensity measurements in the apex (squares), middle (circles), and base (triangles). Graphs **(B,E,H,K)** present intensity changes in the same regions at each time point with respect to the initial values at 0 min. Δ*I* is plotted with a reversed sign (–Δ*I*) so that larger numbers indicate increased dye. *I*_max_ was defined as the intensity change (Δ*I*) at 3 min. Graphs **(C,F,I,L)** show the percentage of intensity change at each time point, in respect to *I*_max_, during injection. **(M)** Intensity changes 3 min after starting the pump in respect to the intensity values at 0 min (*I*_0_). Asterisks indicate levels of significant change in intensity values between 3 and 0 min at each cochlear region. **(N)** Intensity changes from 4 min (time point when the micropipette was removed) until 30 min later. Asterisks indicate levels of significant change in intensity values between 34 and 4 min at each cochlear region. Boxes represent standard deviations of the mean. Pair-sample *t*-test in panels **(M,N)** were used for calculating the *p*-values.

**FIGURE 4:**
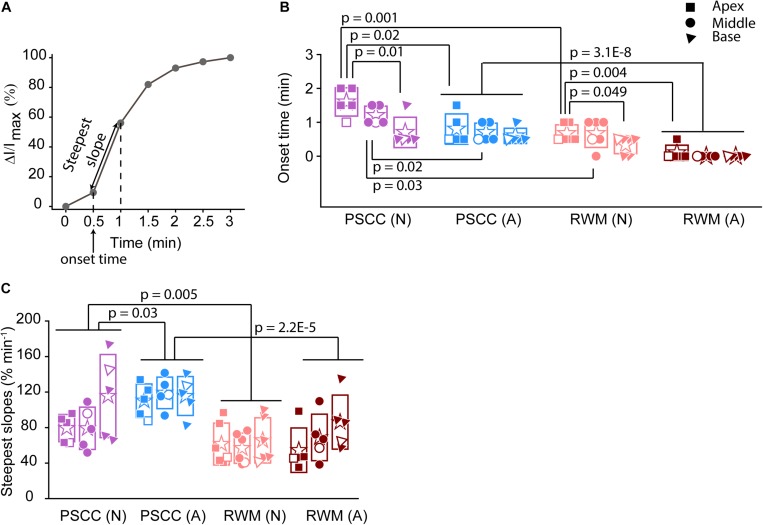
Analysis of normalized intensity changes in the cochlea during the injection period (0–3 min) (summary from data in Figure 3). In each plot N is neonatal and A is adult. Apical is represented by squares, middle by the circles, and base by triangles. Open symbols represent data from the individual examples presented in Figures 3A–L. Graph **(A)** describes the measured parameters presented in panels **(B,C)**. **(B)** Onset time and **(C)** steepest slope obtained from individual animals as shown in the third column of Figure 3. Boxes represent standard deviations of the mean. In panels **(B)** and **(C)** statistical comparisons are done on pooled data showed with horizontal lines. Two-sample *t*-test in panels **(B,C)** were used for calculating the *p*-values.

Data from Figure 2 were analyzed by measuring the average intensity values of pixels within each region of interest (ROI, 50 μm diameter) for each animal. ROIs are demarcated in the last column of Figure 2. Average intensities in the apex, middle, and base were measured from time 0 (starting the injection) until 34 min after starting the infusion. Video examples for each condition are presented in the Supplementary Material S1–S4. The graphs in Figures 3A–L present data analyzed from the movies (Supplementary Videos) for individual animals selected as those with parameters closest to the mean values (presented in Figure 4). Figures 3A,D,G,J show the intensity value decreased significantly for the first 3 min, during dye injection through the PSCC or RWM (Figure 3M). Lower intensity (*I*) values indicate darker regions, meaning the dye is reaching that area. The intensity changes relative to time 0 (Δ*I*) are plotted (as −Δ*I* to more intuitively reflect an increase in dye concentration) for apex, middle, and base in Figures 3B,E,H,K. *I*_max_ was defined as the intensity change (Δ*I*) at 3 min after starting the injection. We interpret intensity changes as equivalent to changes in dye levels. In PSCC injected mice (*n* = 5 for each group of adults and neonates), dye increased at each location for both ages during the entirety of the injection (Figures 3A,B,D,E). In RWM injected neonatal animals (*n* = 6, Figures 3G,H), there was an increase in dye in basal regions with a modest change for mid and very little change for apical regions. Adult RWM (*n* = 5) showed a similar pattern to neonatal, though there was a measurable change in the apical region (Figures 3J,K). These data suggest more dye entry for adult compared to neonatal animals for both injection sites and also that dye reaches the apex more readily for PSCC injections than for RWM injections.

A slight increase in the intensity value was observed at the end of infusion (indicated by the arrow in Figure 3A), correlating with the time when the glass micropipette was removed from the injection site. This increase was not observed in RWM injected animals, likely because the limited amount of dye present at apex and middle ROIs did not allow for the dye reduction detection. The lack of change in the basal region is not due to a lack of dye and perhaps suggests a difference due to injection site.

In PSCC injected animals, the dye level reduced slowly after stopping the infusion (Figures 3A,B,D,E,N). In RWM injected neonatal mice (*n* = 6), after stopping the infusion, apex dye levels were unchanged, there was an increase in dye levels for mid regions and a more robust change at the basal turns, likely indicating a continued progression of dye from the base toward the apex (Figures 3G,H,N). In RWM injected adults (*n* = 5), dye levels increased in all turns within 30 min post injection again suggesting continued dye progression after the perfusion (Figures 3J,K,N).

**FIGURE 5:**
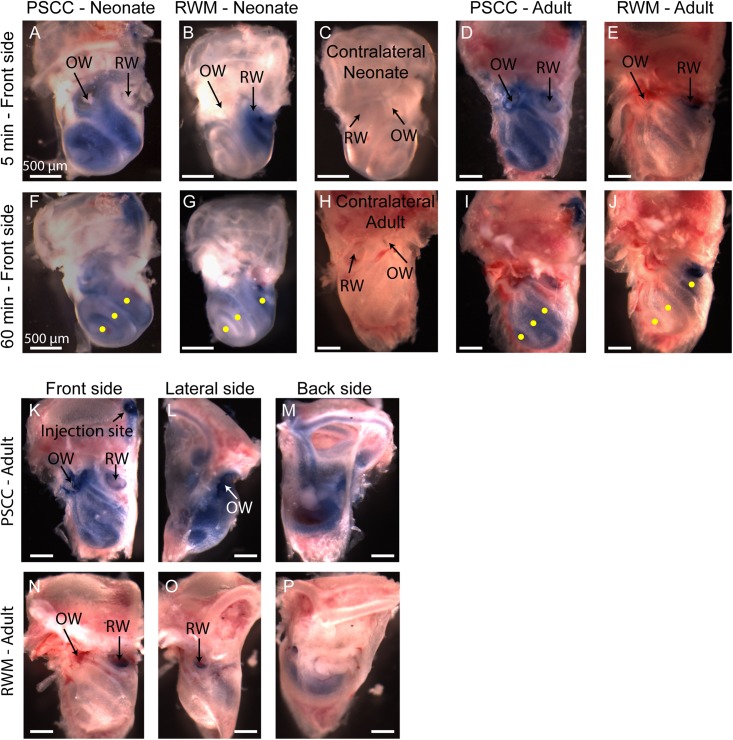
Representative pictures of the mouse cochleae, after injecting trypan blue through RWM and PSCC, at 5- **(A–E)** and 60-min **(F–J)** post injection. The contralateral cochleae of neonatal and adult mice are also presented for comparisons **(C,H)**. Regions of interest (100 μm diameter) where the average intensity values were measured are shown with yellow dots in **(F,G,I,J)**. Front, lateral, and back side of adult mice, 5 min after injecting through PSCC **(K–M)** and RWM **(N–P)**.

**FIGURE 6:**
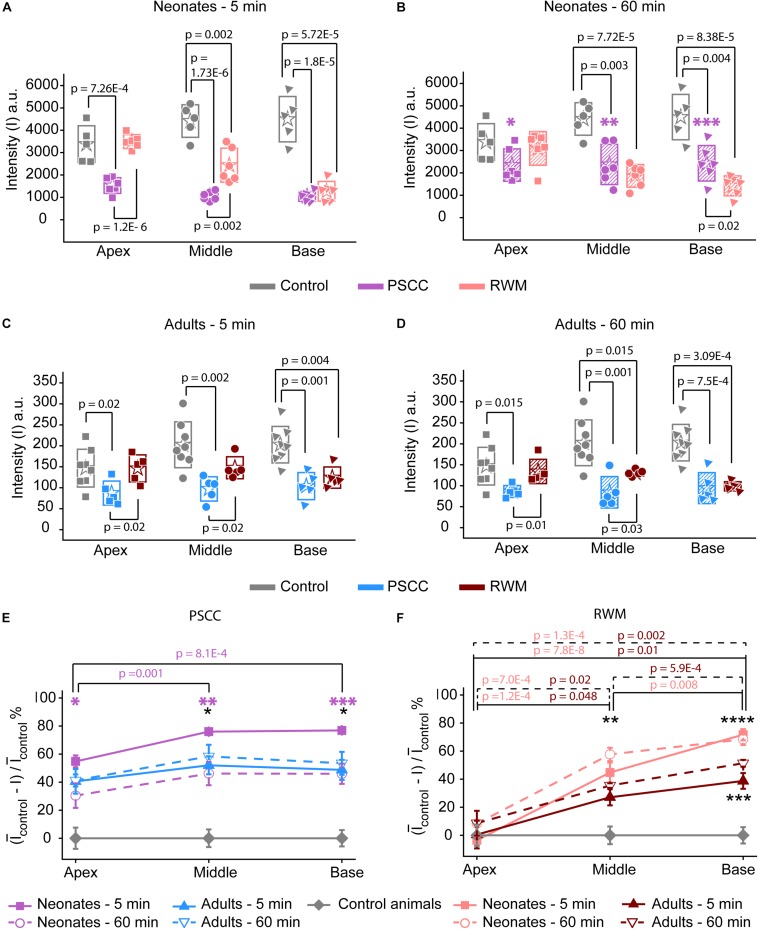
*Ex vivo* assessment of the presence of trypan blue in the PSCC and RWM injected and contralateral cochleae of neonatal mice at 5 **(A)** and 60 min **(B)**, and adult mice, at 5- **(C)** and 60-min **(D)** post injection. The contralateral cochleae of neonatal and adult mice were used as control (non-injected) cochleae. Average intensity values measured within 100 μm regions (shown with yellow dots in Figures 5F,G,I,J) in apex, middle, and base. Measurements in panels **(A,B)** were taken from pictures with TIFF formats (12 bit) and panels **(C,D)** from the ones with JPEG (8 bit). Boxes represent standard deviations of the mean. Percentage of intensity change at each region of cochlea in respect to the control values at both time points for PSCC **(E)** and RWM **(F)** injected animals. Error bars represent standard errors. Purple asterisks in panels **(B)** and **(E)** indicate significant differences between data values at 60 min compared to 5 min at each cochlear region of PSCC injected neonates (^∗^*p* = 0.03, ^∗∗^*p* = 0.006, ^∗∗∗^*p* = 0.002). Black asterisks in panels **(E,F)** indicate significant differences between the data values for neonatal and adult mice (^∗^*p* = 0.006 at 5 min, ^∗∗^*p* = 0.003 at 60 min, ^∗∗∗^*p* = 8.3*E*–4 at 5 min, ^****^p = 0.007 at 60 min). Two-sample *t*-test was used for calculating the *p*-values between groups.

A summary of maximal changes monitored at the end of the injection (from the imaging angle shown in Figure 2) shows a base to apex gradient for PSCC injected neonates and RWM injected animals (Figure 3M). PSCC data lose this gradient in adult, while RWM injections are not different between ages. The relative changes associated with PSCC injection were greater than RWM injections (Figure 3M); however, the orientation of cochlea during *in vivo* imaging provides a better view for monitoring the dye progression in PSCC injection compared to the RWM so we performed additional experiments to obtain more equivalent views (see Figures 5, 6 for more direct comparisons at different orientations).

Dye distribution was evaluated 30 min after stopping the infusion relative to the time immediately after removing the pipette (Δ*I*_30__min_). The majority of PSCC mice showed a reduction in dye accumulation, as indicated by positive values at 30 min post injection (Figure 3N). RWM injected mice showed a continued increase in dye accumulation as indicated by negative values meaning more dye present (Figure 3N). These data support the idea that PSCC had uniform distribution early with later time points reflecting diffusion out of the cochlea. In contrast, RWM injections showed less dye within the cochlea; the existing dye distributed more uniformly over the following 30 min.

To evaluate the kinetic differences between modes of injection independent of absolute concentrations we normalized data to the time point immediately before termination of the injection (Figures 3C,F,I,L). The presented examples demonstrate time delays and rate differences in dye progression between modes of delivery and age. The dramatic differences in dye level are not included in these plots but rather simply an indication of the timing differences, summaries of which are presented in Figure 4. The measured parameters are depicted in Figure 4A. Data from the individual examples shown in Figures 3A–L, are indicated with open symbols in Figures 4B,C. The onset time is defined as the first time point where the steepest rate of change for dye accumulation occurs (Figure 4A). Figure 4B summarizes changes in onset time between groups. A delay in onset time was observed between regions for neonatal animals, being most delayed in the apex, regardless of delivery mode. Adult animals did not show intracochlear differences in onset time. PSCC injections were more delayed than RWM injections in all groups as might be predicted from the longer distance between the injection site and cochlea in PSCC compared to RWM delivery. The age difference may simply represent the change in size of the inner ear reducing resistance to flow in adult mice.

The intensity change over time was not linear for any age or delivery mode (Figures 3C,F,I,L). To compare rates of change we simply used the steepest slope as described in Figure 4A, for each experimental group. Given that these measurements are during perfusion a common slope is predicted that basically relates injection rate to cochlea properties. Reductions in this rate suggest that flow is bifurcating or that different resistances are encountered. That is, if the dye splits into flow in multiple directions the rate will be reduced for either pathway. Results of this analysis are shown in Figure 4C. The steepest slope had larger values in the PSCC injected animals (both neonates and adults) compared to the RWM injected ones. The PSCC injected adult mice had steeper slopes compared to the neonatal ones; but no significant difference in the slope was observed between the neonatal and adult mice injected through the RWM. The slope difference between ages is likely a result of a reduced resistance to flow in the adult animal. The steeper slope with PSCC injections suggests higher levels of dye are entering the cochlea from this site of injection at each time unit. All biological paths from the PSCC injection lead through the cochlea while the RWM injection can bifurcate to the cochlea aqueduct prior to distributing through the entire cochlea. This simple difference likely accounts for the difference in dye distributions for the two injection sites.

A problem with the *in vivo* imaging is that orientation of the cochlea makes it more difficult to assess distribution with RWM injections. To further assess diffusion post injection and to better visualize dye distributions throughout the cochlea a separate set of experiments was performed where the brains and both cochleae were obtained at 5- and 60-min post injection for each experimental group. This approach allows us to evaluate dye distribution *ex vivo* within the cochlea from angles that were not accessible in the *in vivo* images. A representative cochlea from each group of experiments (*n* = 6 and *n* = 5 in each group of neonatal and adult cochleae, respectively) is shown in Figure 5. No dye was detected in any contralateral cochleae. It is clear from the images that the PSCC injections achieved more uniformly distributed dye than did the RMW injections. To better assess the dye pathway with RWM injections and to investigate uniformity of distribution, images were obtained from three perspectives at 5 min post injection (Figures 5K–P). These data demonstrate that PSCC has high dye levels throughout the cochlea and even SCCs. In contrast, RWM injections show dye in the basal areas with less distribution to apical regions. In addition, these data suggest that dye intensity differences may in part be due to the dye going elsewhere. This conclusion is supported also by the reduced steepest slopes described in Figure 4.

The intensity values from regions with 100 μm diameter at apex, middle, and base were measured for each cochlea as indicated by yellow dots in Figures 5F,G,I,J. Immediate dissection and inspection of cochlea following the end of PSCC injection (about 5 min post injection) show all cochlea regions were significantly darker than non-injected ones, at both ages (Figures 6A,C). In contrast, RWM injected animals showed a steep gradient where only base in adults and base and middle in neonates were significantly darker than the non-injected cochleae at the 5-min time point (Figures 6A,C).

As shown in Figures 6A,C, the dye was highest at the basal turn of PSCC or RWM injected cochlea, ∼5 min after injection, and values did not differ from each other at either age (*p* = 0.3). In contrast, apical measurements in neonates show dye levels higher for PSCC injected cochleae than RWM injected cochleae (*p* = 1.2*E*−6) while neonatal RWM injected cochleae were not different from control (*p* = 0.7). In adults, apical values in PSCC remained different from RWM injected values (*p* = 0.02) but apical values in RWM are again not different from control (*p* = 0.98).

One hour after injection, dye levels in the PSCC injected neonatal cochleae were significantly reduced in all regions compared to 5 min after injection (Figures 6B,E, see asterisks). Adult animals showed no difference in the dye levels for PSCC injections for any region compared to the 5 min time point (Figures 6C–E). No significant difference in the cochlear dye levels was observed in RWM injected neonatal (Figures 6A,B,F) or adult (Figures 6C,D,F) for 1 h compared to 5 min after injection, suggesting dilution was not happening with this mode of injection at either age.

The percentage of intensity change at each ROI in respect to the control values is summarized at early (Figure 6E) and late (Figure 6F) time points for PSCC and RWM injected animals, respectively. For simplicity, the control data from neonatal and adult mice are combined. The dye distribution in neonatal animals presented a gradient from base to apex, after PSCC injection at the 5-min time point (Figure 6E) but not at the 60-min time point. This gradient did not exist in the PSCC injected adults at either time points. A steep gradient in dye distribution at both 5 and 60 min was observed after RWM injection in both neonatal and adult mice (Figure 6F).

**FIGURE 7:**
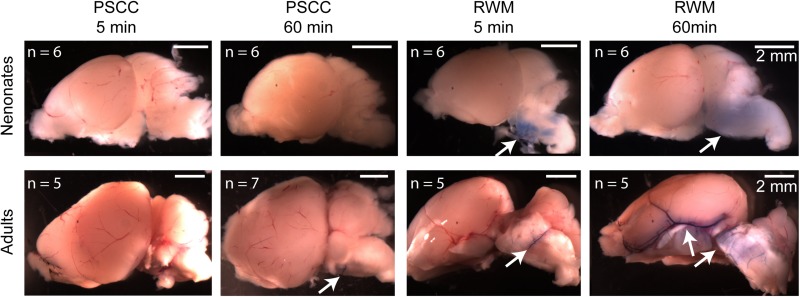
Representative brain pictures of the neonatal and adult mice injected with trypan blue through PSCC or RWM, 5 and 60 min after injection. Number of evaluated brains in each group of experiments is indicated. The arrows point to the regions where the dye was observed.

One possibility is that part of the dye injected through the RWM travels through the cochlea aqueduct which is located very close to the injection site. In contrast, the PSCC injection site is at the opposite end of the cochlear perilymphatic space, so that during the injection perilymph will be pushed through the cochlea aqueduct while dye enters the cochlea. We inspected the brains of injected animals as a proxy for dye traveling through the cochlea aqueduct. Brain images from each group of injected animals are shown in Figure 7. No dye was detected in the brain of the neonatal mice injected through PSCC (at 5 or 60 min, *n* = 6 per group). However, trypan blue was detected in 5/6 neonatal mice brains injected through RWM at both 5 and 60 min supporting the idea of dye travel through the cochlea aqueduct. In PSCC injected adult mice, no dye was detected in the brain 5 min after injection, but small traces of trypan blue were observed in four out of seven adult mice 1 h after injection, suggesting travel through the cochlea aqueduct post injection. The dye was also detected in all adult mice brains injected through RWM at both time points (*n* = 5 for each). Thus, these data are consistent with the hypothesis that RWM injections lose dye through the cochlea aqueduct more readily than PSCC injections.

### Different Substances Have Different Distributions After PSCC Injection

**FIGURE 8:**
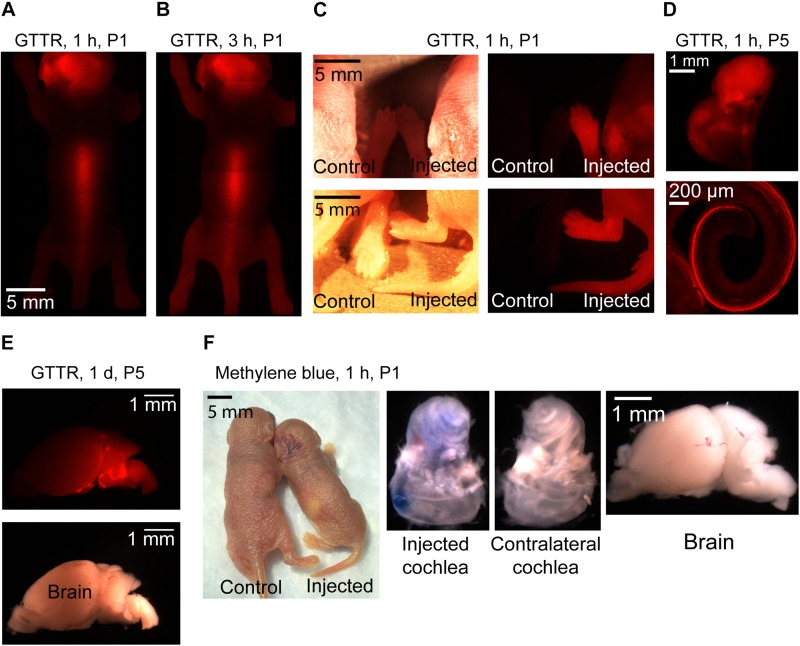
Representative pictures of GTTR and methylene blue distribution in neonatal mice after 1 μl injection into the left PSCC. *n* = 3 for all experiments except GTTR injection to P5 mice and evaluation at 1-h time point (*n* = 4). GTTR was observed through the skin of the animal, 1 **(A)** and 3 h **(B)** after injection. **(C)** Comparing injected and non-injected pups, 1 h after GTTR injection. The bright field and fluorescent images are shown in left and right panels, respectively. **(D)** GTTR in the injected cochlea, 1 h after injection to a P5 pup. Otic capsule and SCCs are shown in the top. Organ of corti is shown in the bottom. **(E)** Brain of the GTTR injected pup, 1 day after injection at P5. The bright field and fluorescent images are shown on the top and bottom images, respectively **(F)** 1 h after methylene blue injection to P1 pup.

It has previously been suggested that the chemical composition of the injected compound can affect distribution within the ear (Nomura, 1961; Salt and Plontke, 2018). In order to investigate the potential variations in compounds progression in the cochlea following the same mode of delivery and using the same injection parameters, two other compounds were tested. We injected GTTR and methylene blue via PSCC in neonatal animals as described above. The results are shown in Figure 8. Methylene blue presented very similar to trypan blue. One hour after injecting methylene blue into the PSCC of P1 mice, no dye was visible through the skin, and after dissection, no trace of dye was detected in the brain or contralateral cochlea (Figure 8F). However, GTTR distribution was starkly different. One hour after injecting GTTR into the left PSCC of mice at P1, it was detected through the skin of the animals (Figure 8A). Three hours after the injection, the drug was still visible in different parts of the body through the skin (Figure 8B). The fluorescent pictures of a mouse’s paws and tail, 1 h after PSCC injection of GTTR is shown in Figure 8C, in comparison with the non-injected pup. GTTR was also injected to P5 mice through PSCC. One hour after the injection, the drug was visible through the whole cochlea, and most of the inner and outer hair cells (Figure 8D). Figure 8E shows that GTTR was also detected in the brain of the P5 injected pups, 1 day after PSCC injection. For this level of distribution, the GTTR must be able to access the blood supply by crossing membranes within the inner ear. These experiments highlighted the fact that in addition to the injection parameters (e.g., volume, flow rate), mode of delivery (e.g., PSCC, RWM), and resistance to the flow within the cochlea that can influence the distribution patterns within the inner ear, under the same conditions, different molecules can propagate differently within the cochlea and whole body.

## Discussion

**FIGURE 9:**
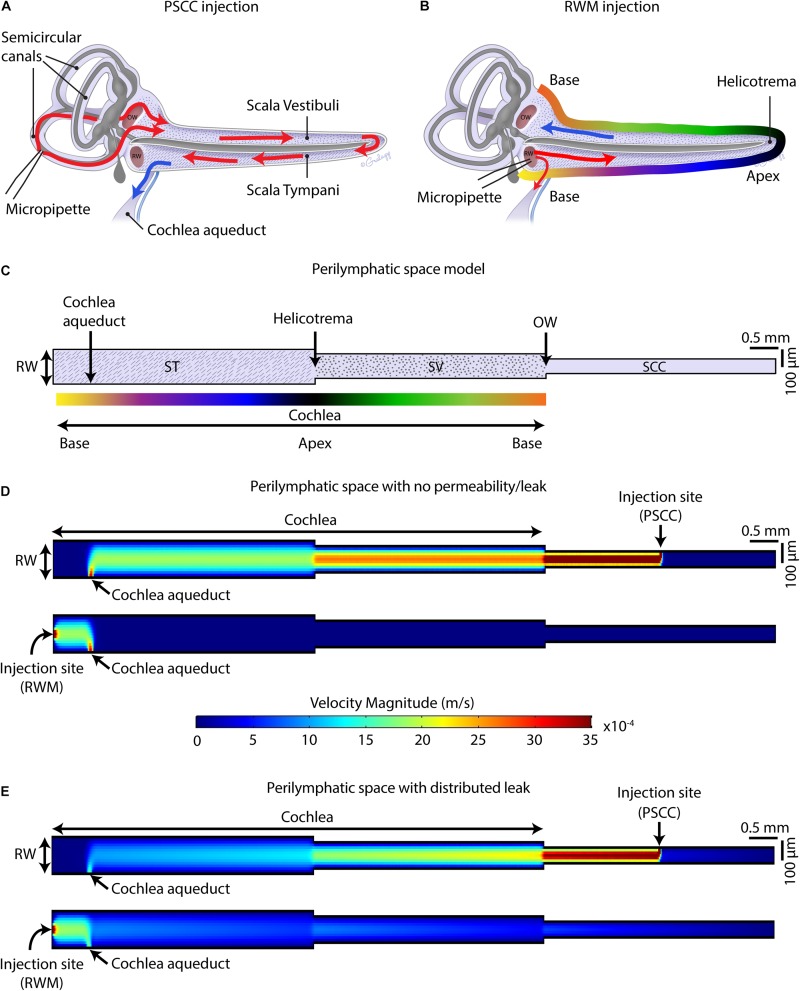
Schematic of mouse cochlea in uncoiled configuration. Pathways of dye distribution in PSCC **(A)** and RWM **(B)** injections are shown in red. Blue arrows show the flow direction of perilymph during injection. **(C)** 2D geometry of the perilymphatic compartment applied for computational modeling of the flow during injections. Results of computing velocity magnitude along the perilymphatic compartment with **(D)** and without **(E)** permeability or leak in the walls. Injection sites (PSCC on the top and RWM on the bottom) were the fluid inlets and cochlea aqueduct was defined as the outlet.

Over the past decade, RWM and PSCC injections are more commonly used for delivering compounds into the inner ear delivering viral vectors for genetic manipulations. Our data set compares PSCC and RWM delivery in neonatal and adult mice using comparable technologies. Identifying and characterizing parameters that modulate drug distribution in the cochlea could help better understand fluid regulation within the inner ear and aid in developing more effective delivery methods for research and therapeutic purposes. Our data provide information on the route of dye flow upon RWM and PSCC injections (Figure 9), the resistance to the dye flow at different ages, and the spread of the dye inside the injected cochlea, to the contralateral cochlea and brain within the first hour after injection.

Our data suggest that PSCC is a better route for drug delivery to the cochlea compared to RWM delivery for several reasons. First, at least in mice, the surgical approach is simpler and the likelihood of damage to middle ear is reduced compared to RWM injection. Second, higher levels of dye reached the cochlea. Third, the dye was more uniformly distributed throughout the cochlea with PSCC injections. And fourth, the dye was more restricted to the inner ear where the injection took place and under these conditions did not reach the brain within the first hour after injection. Our hypothesis for the differences is that the cochlea aqueduct position relative to the injection site is regulating how much dye reaches the cochlea.

Figures 9A,B summarize the hypothesis generated from the data collected in this study. In PSCC injections (Figure 9A), the dye first traveled through the SCC and then entered the basal turn of the cochlea via the SV consistent with previous reports (Salt et al., 2012a). Therefore, the compounds first flow through the SV toward the helicotrema, and then continue through the ST toward the cochlea aqueduct/RWM region. In this direction, the cochlear aqueduct can act as a release valve allowing perilymph to flow and be replaced by the injected volume. Our data support a previous report from guinea pig data suggesting PSCC injection provides a more uniform cochlea distribution of drugs (Salt et al., 2012a). In RWM injection (Figure 9B), the dye directly entered the cochlea through ST. At the end of infusion, a sharp gradient in the dye distribution from base to apex was observed at both ages. This is also in agreement with the results reported by Plontke et al. (2016) who showed a basal–apical gradient in the concentration of fluorescein after RWM injection to guinea pigs, and other groups who tested gene expression in mice cochlea by injecting viral vectors through the RWM, observing lower gene expression in the apex compared to the base (Askew et al., 2015; Chien et al., 2015; Yoshimura et al., 2018). We also measured a lower level of dye intensity changes 1-h after injection within the cochleae of RWM injected neonatal animals compared to the PSCC injected ones.

RWM injection has flow direction from the vestibule end of the ST, where the cochlea aqueduct may act to shunt flow (dye) away from the cochlea, toward the brain, thus reducing dye entry into the cochlea. The presence of dye in the brain of animals injected through RWM supports this hypothesis. Transduction in the contralateral ear and brain after RWM injection of viral vectors has been reported in the literature (Landegger et al., 2017), while no gene expression in the contralateral ear was reported after PSCC injection of viral vectors into the cochlea (Kawamoto et al., 2001) also consistent with the above hypothesis.

Monitoring of dye progression within the cochlea provides a direct investigation of fluid dynamics but it is not without limitations. One limitation is that the bony capsule in adult mice is thicker than the cartilaginous otic capsule in neonates, making absolute comparisons of intensity between ages untenable. Evaluating dye distributions *ex vivo* did provide the needed resolution to probe adult cochlea dye distributions more directly. The dye intensity was large enough to be detected through the bone so relative changes and timing in dye distribution were compared within each age group. A second major limitation is that the imaging plane provides different volumes for apex, middle, and base so that data can be misleading regarding absolute differences between regions. This problem is compounded by the injection sites being on opposite ends of the perilymphatic space so that imaging orientation can bias dye tracking depending on injection site. The *ex vivo* experiments allowed viewing of cochlea from multiple orientations and so allowed us to interpret dye changes more convincingly. A third limitation in these comparisons is the difference in anesthesia. Hypothermia as an anesthetic in neonatal mice could affect dye distribution indirectly for neonatal animals. It is possible that the difference in apparent resistance is in part due to the temperature difference, in addition to the size difference between adult and neonatal. However, the expected change in viscosity is about threefold which when investigated in the model described below had little effect on the results. Our measurements occur either during perfusion or for at most 1-h post perfusion which does not allow enough time for diffusion to be effective. It is possible that changes in volume or wall mechanics alter the resistance to flow in a temperature dependent way that we cannot account for and so some caution should be taken in comparing across modes of anesthesia. In general, though the major findings happen within groups. The sampling rate due to the small changes in intensity were relatively slow and so it is possible that subtle differences in dye kinetics could be missed. Overall the fundamental conclusions presented are consistent with previous studies and well supported by the data presented here.

Using a FEM simulation, we generated a simplified model to demonstrate the feasibility of our hypothesis that the nearness of the injection site to the cochlea aqueduct dictates the pattern of dye distribution within the cochlea. Figure 9C presents the model geometries which represent an unrolled perilymphatic space including the ST and SV and SCCs. The color gradient in Figure 9C is also represented in Figure 9B as a tool to understand how the cochlea was unrolled. Also included in the model structure are the cochlear aqueduct and the two injection sites. The simulation was run on two models with the same geometry but different boundary conditions. The first model assumed that the perilymphatic space is an isolated compartment with no permeability or leak. The only flow inlet was the injection site, and the only outlet was the cochlea aqueduct (Figure 9D). The second model assumed that in addition to the cochlea aqueduct acting as an outlet, a distributed leakage occurred within the perilymphatic space during the injection (Figure 9E). The figure presents velocity as an indicator of flow and is expected to be a correlate of the dye distributions measured during the injection. The direction of the flow was from injection site to the cochlea aqueduct, no intrinsic cochlea flow was included. Unrolling the cochlea shows the clear difference in location of the injection sites with the RWM injection reaching the cochlea aqueduct before the cochlea and the PSCC injection reaching the cochlea before the cochlea aqueduct. This fundamental difference is postulated to explain the difference in dye distribution. In PSCC injection (Figure 9D, top), a relatively uniform high velocity is distributed along the SV and ST during the injection. In contrast in RWM injection (Figure 9D, bottom), the high resistance within the cochlea, due to it being a closed system, the velocity distribution is limited to the area between the RW and the cochlea aqueduct. This example is meant to show the importance of the outlet position relative to the cochlea and injection site. In the second model where a perilymphatic leak was included, the velocity pattern was more like the dye patterns observed experimentally. The basal to apex gradient with RWM injection as well as the lower velocity values in the cochlea due to the bifurcation with the cochlea aqueduct were observed. The PSCC injection resulted in higher average cochlear velocities and more uniformity throughout the cochlea than did RWM injections. This model is used simply to illustrate the validity of our hypothesis and a great deal more detailed information is needed to make it more physiologically realistic.

Our hypothesis supports a significant role for the cochlea aqueduct in dictating flow through the cochlea. As patency of the cochlea aqueduct is potentially variable, particularly between species, it will be important to assess cochlea aqueduct in human if therapeutic approaches are being considered. Gopen et al. (1997) examined temporal bones in human and showed that 93% of the cochlea aqueducts in 101 samples were not completely obstructed (i.e., in 34% the central lumen was patent throughout the length of aqueduct and in 59% the lumen was filled with loose connective tissue). The similarity of data between guinea pig and mouse supports a similar role in fluid regulation for the cochlea aqueduct. The functional role of the cochlear aqueduct is unclear but is postulated to serve as a pressure regulator for the inner ear (Carlborg et al., 1982) and its role in modulating drug delivery must be considered.

Despite providing a uniform distribution of the compounds with PSCC injections, gene expression within the cochlea induced by delivering viral vectors through the PSCC has resulted in higher rates of transduction in the apex compared to the base (Isgrig et al., 2019). This discrepancy between uniformity of the viral vector’s distribution and level of gene expression along the cochlea prompted further investigations to search for factors other than the viral vector distribution that can be responsible for gene expression non-uniformity. We found no difference in elimination times along the cochlea for dyes when compared between injection sites within 1-h after injection. Since we do not know the time frame of elimination, it remains plausible that the transduction gradient is a result of exposure time to the virus. It is also possible that like GTTR, the viral vectors’ distribution is influenced by permeability properties and not driven by accessibility via the injected flow. Another possibility though is that there are tonotopic differences in how the virus interacts with sensory and supporting cells. Our data simply show that dye distribution patterns do not directly mimic expression patterns from viral transduction.

Our experiments suggest a higher resistance to fluid flow within the cochlea of younger animals. A longer onset time to steepest rate of change in dye concentration and smaller rate of intensity change in neonatal animals compared to the adults during injection are observations supporting this conclusion. The higher resistance is likely due to the fluid pathways with smaller dimensions in younger animals. It might also be a function of cochlea aqueduct patency with reduced patency modulating total resistance to flow. Thus, animal age can also influence the rate of delivery.

In RWM injections, where the injection site is near the opening of the cochlea aqueduct into the ST, more dye was observed in the brain of animals compared to PSCC injections. Our results are in agreement with the observations of Akil et al. (2019) who injected a biological dye into the RWM of neonatal mice, and within a few minutes detected the dye in the brain and spinal cord of the mice (Akil et al., 2019). Kaupp and Giebel (1980) also reported the appearance of lyophilized rhodamine in the subarachnoidal space after direct application of the substance into the tympanic perilymph near the RW (Kaupp and Giebel, 1980). Gene expression was also observed in the brain after RWM delivery of viral vectors into the cochlea (Landegger et al., 2017). These data support the idea that the cochlear aqueduct can provide a pressure sensitive pathway to the brain. Compounds shunting to the brain, which happens more in older animals and with RWM injections, reduces the dose of injected compounds received by the cochlea. This shunting can also explain the difference in steepest slopes obtained between injection sites. Together these data support the idea that the cochlear aqueduct serves as a pressure release valve and must be taken into account when trying to deliver compounds to the cochlea. Recent data (Yoshimura et al., 2018) demonstrated uniform delivery to the cochlea with RWM injection if a hole was first created in the PSCC. This hole likely provides an additional outlet for the flow that directs more fluid toward the apex, OW, and SCCs successively. Thus, this is very much in accord with a release valve role for the cochlea aqueduct.

Contrary to the trypan blue and methylene blue experiments, injection of GTTR into the PSCC of neonatal mice resulted in distribution of the compound throughout the body within 1 h. This supports the idea of Nomura that cochlear capillaries have different permeabilities based on chemical structure (Nomura, 1961). It also suggests that the chemical structure of compounds may dictate their permeation across the inner ear membranes in order to access the blood supply. Careful consideration of the chemical properties of a given compound will be needed when local inner ear drug delivery is the goal.

## Conclusion

Data presented here are in good agreement with work from others suggesting that the mode of drug delivery (i.e., RWM or PSCC injection) will alter the distribution of compounds within the cochlea. PSCC injection provides a more uniform distribution throughout the cochlea regardless of age. PSCC injections also result in less dye accessing brain. Flow resistance decreases with age but remains an impediment to distribution. Data are consistent with the cochlear aqueduct serving as a pressure release valve that can help to uniformly distribute drugs when injected through the PSCC but can inhibit drug delivery to the cochlea by diverting flow (drug) when injected via the RWM. And finally, the chemical nature of the delivered compound will affect the drug spread based on its ability to cross membranes and potentially enter the blood stream.

## Data Availability Statement

The datasets generated for this study are available on request to the corresponding author.

## Ethics Statement

The animal study was reviewed and approved by the Stanford APLAC.

## Author Contributions

ST, MS, and KA: data collection. ST, MS, and AR: data analysis, interpretation, and figure production. ST and AR: method development and writing – original draft. ST: surgical approach, injection technique, anesthesia and recovery for neonatal animals. ST: computational modeling. MS: method development including micropipette production, dye selection, and injection technique. KA: surgical approach, injection technique, anesthesia and recovery for adult animals. AR: resources, supervision, and funding acquisition. ST, MS, KA, and AR: writing – review and editing.

## Conflict of Interest

The authors declare that the research was conducted in the absence of any commercial or financial relationships that could be construed as a potential conflict of interest.

## Footnotes

1 https://www.dvdvideosoft.com/

2 https://fiji.sc/

## Supplementary Material

VIDEO S1 Video example of trypan blue progression in neonatal mouse inner ear during PSCC injection.

VIDEO S2 Video example of trypan blue progression in neonatal mouse inner ear during RWM injection.

VIDEO S3 Video example of trypan blue progression in adult mouse inner ear during PSCC injection.

VIDEO S4 Video example of trypan blue progression in adult mouse inner ear during RWM injection.
